# Assessment of *ESR1*, *PGR*, *ERBB2*, and *MKI67* mRNA in Hormone Receptor‐Positive Early Breast Cancer: A Cross‐Sectional Study

**DOI:** 10.1002/hsr2.71062

**Published:** 2025-07-15

**Authors:** Hamid Rezvani, Shayan Forghani, Arman Forghani, Fatemeh Mahdavi Sabet, Atieh Akbari, Sanaz Tabarestani

**Affiliations:** ^1^ Department of Hematology‐Oncology Shahid Beheshti University of Medical Sciences Tehran Iran; ^2^ Cancer Research Center Shahid Beheshti University of Medical Sciences Tehran Iran

**Keywords:** breast cancer, estrogen receptor, HER2 receptor, ki67, progesterone receptor

## Abstract

**Background and Aims:**

Hormone receptors are expressed in 70% of breast cancers and are the major biomarkers for tailoring treatment in early‐stage breast cancer. In clinical routine, immunohistochemistry (IHC) is used to assess estrogen receptor (ER), progesterone receptor (PR), HER2, and Ki67 protein expression. However, IHC procedure is challenged with pre‐analytical and analytical variability. Pathologist interpretation of IHC results can vary, and discordant results between local and reference laboratories have been reported. Using mRNA‐based tests may be a more robust, reliable, and standardized method to assess these important breast cancer biomarkers. This study aimed to assess the concordance between real‐time PCR and IHC results.

**Methods:**

In this study, we analyzed 178 early‐stage hormone receptor‐positive breast tumors. IHC for ER, PR, HER2, and Ki67 had been previously performed for the study samples at local laboratories. For samples with HER2 IHC score 2+, Fluorescence In Situ Hybridization was performed. *ESR1* (encoding ER), *PGR* (encoding PR), *ERBB2* (encoding HER2), and *MKI67* (encoding Ki67) mRNA expression were determined using TaqMan gene expression assays.

**Results:**

The overall concordance between mRNA expression results and their corresponding IHC markers was 95.9% for *ESR1*/ER, 79.3% for *PGR*/PR, and 100% for *ERBB2*/HER2. There was a moderate correlation between MKi67 mRNA values and Ki67 IHC. ESR1 expression was significantly lower in tumors of younger patients (*p* < 0.001). No statistically significant correlation between age at cancer diagnosis and ER IHC was identified. Higher *ESR1* and *MKI67* mRNA expression was associated with worse pathological characteristics.

**Conclusions:**

PCR‐based classification of breast tumors in a central laboratory may be used to confirm the available IHC results performed at local laboratories and add valuable information for patient management. mRNA‐based biomarkers may be promising for more standardized breast cancer management.

## 1 Introduction

Breast cancer is the leading female neoplasm worldwide. Breast cancer is considered a heterogeneous disease, and it is categorized into different molecular subtypes based on different gene expression patterns. Hormone receptors are expressed in about 70% of breast cancers and are the major biomarkers for tailoring treatment in early‐stage breast cancer [1]. The human epidermal growth factor receptor 2 (HER2) is overexpressed in 10%–15% of breast cancers and is indicative of aggressive cancer [2]. Ki67 is a marker of the proliferation of cancer cells and is widely used in clinical practice. However, many international guidelines do not consider ki67 as a reliable marker in clinical routine due to the absence of reproducibility [3, 4].

In clinical routine, immunohistochemistry (IHC) is used to assess the status of estrogen receptor (ER), progesterone receptor (PR), HER2, and Ki67 protein expression. However, IHC procedure is challenged with pre‐analytical and analytical variability, including fixation delay and duration, sensitivity of antibody clones, and interobserver variability with regard to staining intensity [5]. Pathologist interpretation of IHC results can be different, and many studies have shown that the results of local and reference laboratories are discordant. De Duenas et al. reported a concordance rate of 92% for ER, 78% for PR, and 83% for HER2 between local and reference laboratories [6]. In the study reported by Orlando et al., the concordance rate for ER, PR, HER2 were 82%, 86%, and 73%, respectively [7]. Standardization of pre‐analytical, analytical, and post‐analytical steps of IHC procedure is critical to improve the reproducibility and accuracy of results: pre‐analytical variables (including fixation time, fixative type, Ph and buffer, and paraffin embedding temperature), analytical variables (including sensitivity and specificity of antibody, positive and negative controls, and staining using automated platforms), and post‐analytical variables (guideline recommended scoring criteria) [8].

As multiple studies have shown association between quantitative measurement of *ESR1* and *ERBB2* mRNAs (which encode ER and HER2 proteins) and clinical outcome on tamoxifen and trastuzumab [9, 10, 11], the use of mRNA‐based tests may be a more robust, reliable, and standardized method to assess these important breast cancer biomarkers [12].

We designed the present study to evaluate the concordance between IHC and quantitative reverse transcription polymerase chain reaction (qRT‐PCR) for the analysis of ER, PR, HER2, and Ki67 in breast cancer specimens that are defined as hormone receptor‐positive by IHC in local laboratories.

## 2 Methods

### 2.1 Sample Selection

We analyzed 178 hormone receptor‐positive breast cancer patients treated in the Cancer Research Center, Shohada Tajrish Hospital, and associated clinics from February 2022 to December 2024. The inclusion criteria were female patients older than 18 who were diagnosed with invasive ER‐positive, PR‐positive, and HER2‐negative non‐metastatic breast cancer with ductal carcinoma histology. Samples with ER and PR IHC ≥ 1% stained cells were defined as ER‐ and PR‐positive, respectively [13]. It was necessary that the FFPE (formalin‐fixed paraffin‐embedded) sample of the primary tumor of each patient be available. Exclusion criteria were receiving neoadjuvant chemotherapy, lack of invasive breast tumor in the available FFPE block, ER‐, PR‐, or HER2‐negative breast tumors, or breast cancer histologic types other than invasive ductal carcinoma. This study was approved by the institutional review board (IR.SBMU.CRC. REC.1403.007), and all patients signed the informed consent form according to our Institutional Review Board recommendations. IHC for ER, PR, HER2, and Ki67 had been previously performed for the study samples at local laboratories. For samples with HER2 IHC score 2+, Fluorescence In Situ Hybridization (FISH) was performed.

## 3 RNA Extraction and Purification

For each sample, a slide was stained with hematoxylin and eosin (H&E) to identify the invasive cancer and estimate the invasive cancer percentage. For samples with less than 50 percent invasive tumor content, macrodissection was performed. RNA was extracted from two 10‐µm sections of FFPE tissue with the Qiagen RNeasy FFPE Kit (QIAGEN, Hilden, Germany). Qubit RNA High Sensitivity kit (Thermo Fisher Scientific, CA, USA) was used to quantify the extracted RNA. The purity of the RNA was evaluated using a NanoDrop spectrophotometer (Thermo Fisher Scientific, Oregon, USA). Residual DNA contamination was assessed with TaqMan *ACTB* assay using positive and negative controls. Positive control was DNA extracted from the breast tissue, while negative control was no‐template control. In case of DNA contamination, the RNA was treated with DNase I (DNA‐*free* kit, Thermo Fisher Scientific, Oregon, USA) and assessed again for DNA contamination.

## 4 Reverse Transcription and Real‐Time PCR

cDNA was synthesized from the isolated RNA with random hexamers (Qiagen, Hilden, Germany) using Omniscript RT kit (QIAGEN, Hilden, Germany). We incubated the reaction mixture for 60 min at 37°C. Expression of *ESR1* (encoding ER), *PGR* (encoding PR), *ERBB2* (encoding HER2), and *MKI67* (encoding Ki67) mRNAs were detected using TaqMan gene expression assays (Thermo Fisher Scientific, Pleasanton, CA), normalized to five known reference genes in breast tissue (*ACTB*, *GAPDH*, *GUSB*, *RPLP0*, and *TFRC*) [14] (Supporting Information S2: Table 1).

We performed real‐time PCR reactions in triplicate using QuantStudio 3 (Thermo Fisher Scientific, CA, USA). Amplification reactions were performed with qPCRBIO Probe Mix Lo‐ROX (PCR Biosystems Ltd, London, UK) in a final volume of 20 µL. PCR conditions were 2 min/95°C, and then 40 cycles of 5 s/95°C and 25 s/60°C. We used Quanstudio 3 software to determine gene expression normalized against the mean expression of the five reference genes. The expression of each gene was determined using the delta Ct (cycle threshold) method, $\;∆\mathrm{Ct}\;={\mathrm{Ct}}_{\mathrm{reference}}\;–\;\;{\mathrm{Ct}}_{\mathrm{target}\;\mathrm{gene}}$. Delta Ct cut‐offs for *ESR1*, *PGR*, and *ERBB2* were set at “−3.5”, “−4.5”, and “0.7”, respectively, as previously determined [15, 16, 17, 18].

## 5 HER2 Fluorescence In Situ Hybridization (FISH)

FISH was perfomed using cytocell HER2 dual probe kit (CytoCell, Oxford Gene Technology Inc., UK). Signals were scored in 40 total nuclei from an invasive breast tumor. Samples were considered HER2 amplified on the basis of 2023 ASCO (American Society of Clinical Oncology)‐CAP (College of Pathologists) guideline [19].

## 6 Statistical Analysis

Descriptive statistics was used to analyze patient characteristics. Continuous data were assessed for distribution normality using Kolmogorov–Smirnov test. We performed all statistical analyses against a two‐sided alternative hypothesis and used a significance level of 0.05. Spearman's rank correlation was used to assess the variability in the marker expression results between IHC/FISH and qRT‐PCR. The association between mRNA expression and clinicopathologic features was assessed using *t*‐test for data exhibiting normality and Wilcoxon rank‐sum test for those not normally distributed. We performed statistical analysis using SPSS software, version 27.0.1 (IBM Corp., Armonk, NY, USA) and R Statistical Software version 4.3.1 (Vienna, Austria).

## 7 Results

### 7.1 Patient Characteristics

**Figure 1:**
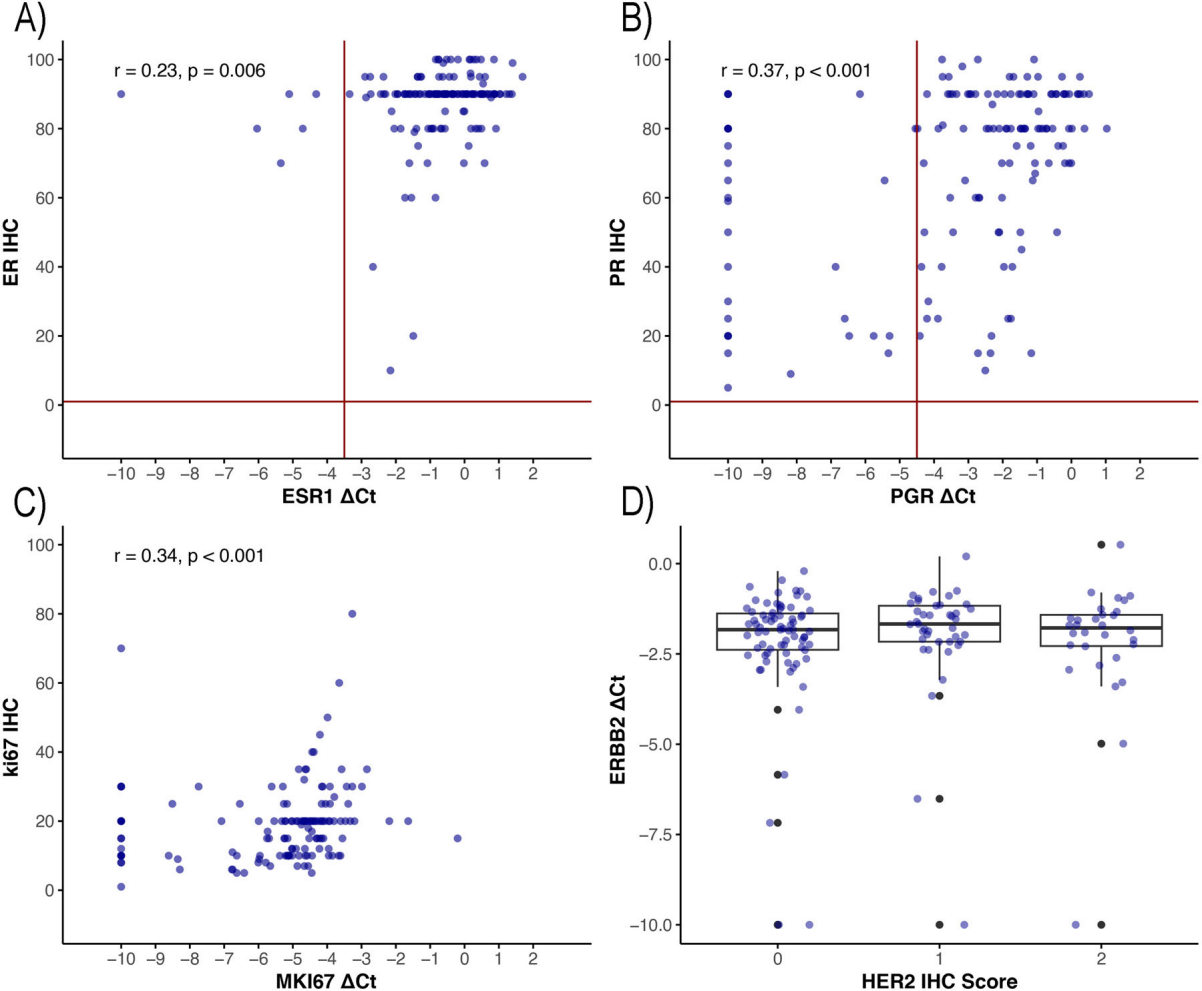
Scatter plots of the distribution of mRNA expression and IHC. (A) *ESR1* mRNA/ER IHC; (B) *PGR* mRNA/PR IHC; (C) *MKI67* mRNA/Ki67 IHC; (D) Boxplot of distribution of *ERBB2* mRNA expression and HER2 IHC. Each dot represents a patient. Abbreviations: Ct, cycle threshold; ER, estrogen receptor; HER2, human epidermal growth factor receptor 2; IHC, immunohistochemistry; PR, progesterone receptor; r, Spearman's rank correlation.

Of the 178 patients identified, 150 fulfilled the selection criteria, and 145 were finally analyzable. Four out of 150 initially included were considered not analyzable because the available FFPE block contained only DCIS (ductal carcinoma in situ) and no invasive breast tumor (*n *= 1), sufficient RNA was not extracted from tumor block (*n *= 1), and FISH signals were not optimal and rejected (*n *= 2). One sample was HER2‐amplified by FISH, so it was excluded from the study (Supporting Information S1: Figure 1). All tumors were surgical specimens, and the FFPE block age range was 1–6 months. A summary of the characteristics of the patients are depicted in Table 1. The median age at breast cancer diagnosis was 49 years (interquartile range (IQR): 42–60 years). One hundred and thirty‐three patients (91.7%) were diagnosed with unilateral and unifocal breast cancer. One out of 145 tumors (0.7%) were ER‐low‐positive (1%–10% stained tumor cell nuclei), and 3 out of 145 tumors (2.1%) were PR‐low‐positive.

**Table 1:** Clinical and pathological characteristics of the patients (*N* = 145).

Variable	No.	%
Age at breast cancer diagnosis median (IQR)	49 (42–60)	16.6
≤ 40	24	57.2
41–59	83	26.2
≥ 60	38	
Tumor focality		
Unifocal	133	91.7
Multifocal	10	6.9
Bilateral	2	1.4
Tumor grade		
1	29	20
2	105	72.4
3	10	6.9
Missing	1	0.7
Tumor size (cm)		
≤ 1	25	17.2
> 1–2	59	40.7
> 2–4	61	42.1
Nodal status		
0	129	89
1–3	13	9
> 3	0	0
Missing	3	2
Stage		
I	83	57.2
II	58	40
III	1	0.7
IV	0	0
Missing	3	2.1
ER IHC nuclei staining		
1%–10%	1	0.7
11%–50%	2	1.4
> 50%	142	97.9
PR IHC nuclei staining		
1%–10%	3	2.1
11%–50%	35	24.1
> 50%	107	73.8
HER2 IHC score		
0	74	51
1	41	28.3
2	30	20.7
3	0	0
Ki67 IHC staining		
≤ 5%	4	2.8
6%–29%	111	76.6
≥ 30%	25	17.2
Missing	5	3.4

Abbreviations: ER, estrogen receptor; HER2, human epidermal growth factor receptor 2; IHC, immunohistochemistry; IQR, interquartile range; PR, progesterone receptor.

## 8 Concordance Between IHC and Real‐Time PCR

The overall concordance between mRNA expression levels of *ESR1*, *PGR*, and *ERBB2* and their respective immunohistochemical (IHC) markers—ER, PR, and HER2—was 95.9% (95% CI: 90.8–98.3%) for *ESR1*/ER, 79.3% (95% CI: 71.6–85.4%) for *PGR*/PR, and 100% (95% CI: 96.8–100%) for *ERBB2*/HER2. 6 out of 145 (4.1%) samples classified as ER‐positive by IHC were ER‐negative by qRT‐PCR. *ESR1* ΔCt values versus percent ER‐positive tumor cells are presented in Figure 1A. Thirty out of 145 (20.7%) samples classified as PR‐positive by IHC were PR negative by qRT‐PCR. The PR IHC percent positivity in the PR IHC/qRT‐PCR discordant samples was significantly lower than the concordant samples (*p* < 0.001). The median of PR IHC percent positivity in the PR IHC/qRT‐PCR concordant samples and discordant samples was 80% (IQR: 66%–90%), and 45% (IQR: 20%–79%), respectively. *PGR* ΔCt values versus percent PR positive tumor cells are presented in Figure 1B.

A comparison of *ERBB2* ΔCt values and HER2 IHC score is shown in a boxplot (Figure 1C). There was a moderate correlation between *MKi67* mRNA ΔCt values and percent positive Ki67 staining tumor cells (*r* = 0.34, *p* < 0.001). *MKi67* mRNA ΔCt values and Ki67 IHC results are shown as a scatter plot in Figure 1D.

**Figure 2:**
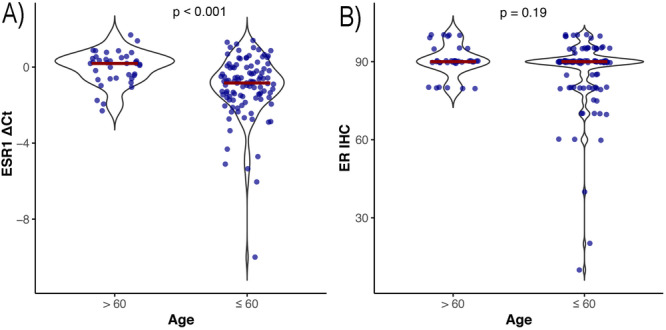
Expression of estrogen receptor mRNA (*ESR1*) and ER protein in older (> 60 years) and younger (≤ 60 years) patients. (A) *ESR1* mRNA; (B) ER IHC. Two‐side Wilcoxon rank‐sum test was used to estimate the *p* values. Each dot represents a patient, the horizontal red line represents the median *ESR1*/ER expression in each patient age group. Abbreviations: Ct, cycle threshold; ER, estrogen receptor; IHC, immunohistochemistry.

*ESR1* mRNA expression was significantly lower in patients under the age of 60 compared to those aged 60 and above (*p *< 0.001) (Figure 2). The correlation between age at cancer diagnosis and ER IHC was not statistically significant (*r* = 0.12, *p* = 0.16). In contrast, we detected a strong correlation between age and *ESR1* mRNA quantity (*r* = 0.39, *p* < 0.001).

**Figure 3:**
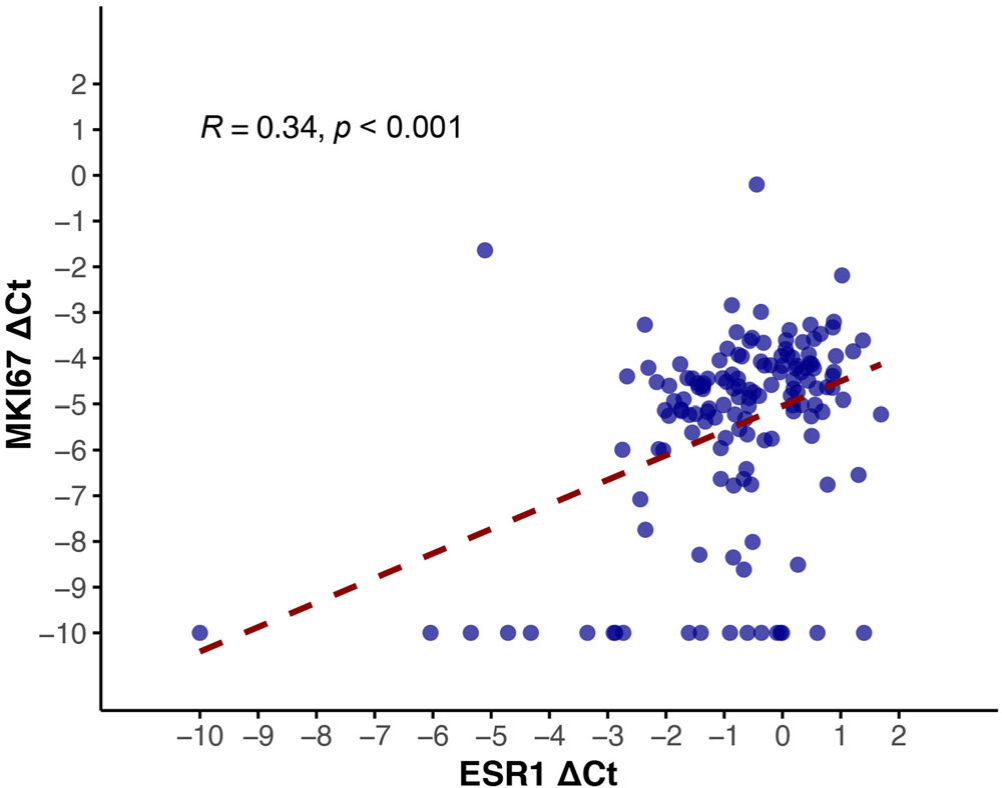
A scatter plot depicting the distribution of tumor *ESR1* and *MKI67* mRNA content. Abbreviations: Ct, cycle threshold; r, Spearman's rank correlation.

**Figure 4:**
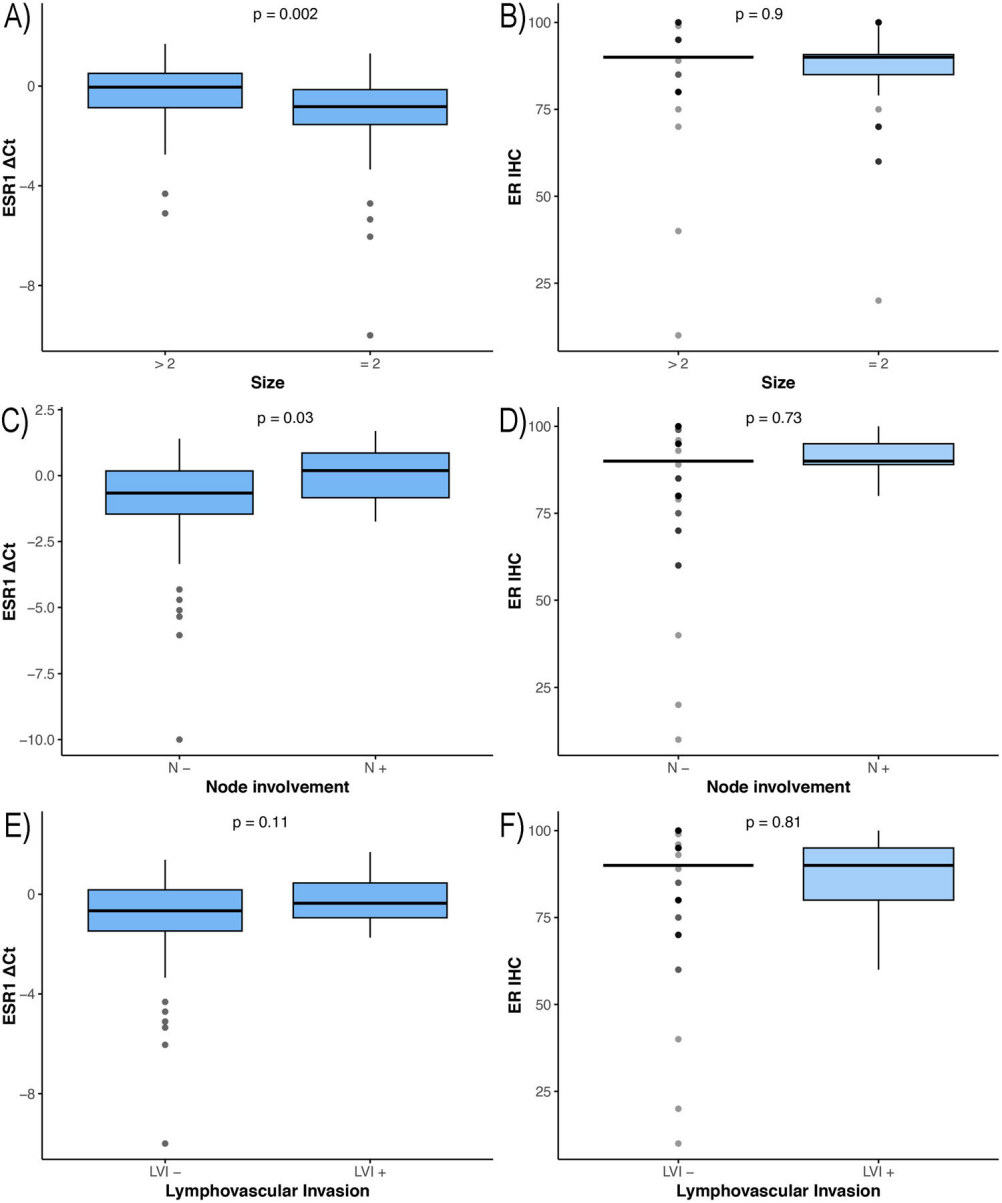
Boxplot of association between ER expression and tumor pathologic characteristics. Two‐side Wilcoxon rank‐sum test was used to estimate the *p* values. (A) *ESR1* mRNA quantity/tumor size > 2 cm or ≤ 2 cm; (B) ER IHC percentage/tumor size > 2 cm or ≤ 2 cm; (C) *ESR1* mRNA quantity/lymph node involvement; (D) ER IHC percentage/lymph node involvement; (E) *ESR1* mRNA quantity/lympho‐vascular invasion; (F) ER IHC percentage/lympho‐vascular invasion.

*ESR1* and *MKI67* mRNA expression also showed a moderate correlation (*r* = 0.34, p < 0.001) (Figure 3). After adjustment for age, node involvement, and lympho‐vascular invasion using partial correlation, *ESR1* and *MKI67* mRNA expression remained moderately correlated (*r* = 0.4, *p* < 0.001). Higher mRNA expression of *ESR1* gene was associated with increased tumor size (*p* = 0.002), more involved lymph nodes (*p* = 0.03), and higher stage (*p *= 0.002). There was no statistically significant association between ER IHC and pathological markers (Figure 4). Higher mRNA expression of *MKI67* gene was associated with increased tumor size (*p* = 0.006), and increased stage (*p* = 0.003). A significant association was detected between increased Ki67 IHC and higher grade (*p* < 0.001).

## 9 Discussion

In the present study, we demonstrated concordance between assessment of *ESR1*, *PGR*, *ERBB2*, and *MKI67* mRNA levels and the IHC analysis of their protein products. IHC offers a visual assessment of protein expression levels within tissue samples and shows the stained antigen distribution in tumor cells and the stroma. However, testing ER, PR, HER2, and Ki67 by IHC has pitfalls. Several guidelines state that 20% of ER, PR, and ki67 results may be incorrect because of differences in pre‐analytical factors, thresholds, and interpretation criteria [4, 13]. In hormone receptor‐positive early breast cancer, these four biomarkers provide important prognostic and predictive information; therefore, their accurate identification is critical.

We found a high agreement between qRT‐PCR and IHC assessment of ER and HER2 (95.9% and 100%, respectively). This is similar to the findings of other researchers. Wilson et al. reported 96% and 97% concordance between mRNA expression and IHC assays for ER and HER2 in clinical breast cancer samples, respectively [20]. In a study on hormone receptor‐positive early breast cancer by Filipits et al., the concordance between ER and HER2 analysis by mRNA expression and IHC were 98.9% and 98.2%, respectively [21]. In a similar study on early breast cancer by Wirtz et al., the concordance between ER and HER2 results by qRT‐PCR and IHC were both 91.8% [22]. In contrast, we identified concordant PR results between mRNA expression and IHC in 79.3% of samples. Other researchers have also described a lower concordance between *PGR* mRNA levels versus IHC. Wilson et al. reported a concordance rate of 81% between PR analysis by mRNA expression and IHC, Filipits et al. reported 89.9%, and Wirtz et al. reported 82.5%. Reduced concordance between PR protein expression and *PGR* mRNA levels has previously been linked to expression values that are near the established cut‐off thresholds, which is similar to our findings [17]. The lower concordance of PR results by IHC and qRT‐PCR in our study may also be due to local IHC results. Several studies have shown a lower concordance between local and central PR IHC results. The reported concordance between central and local PR IHC results has been from 78% to 86% [6, 7, 15].

We identified that breast tumors in patients ≤ 60 exhibit lower *ESR1* gene expression. There was no association between cancer diagnosis, age, and ER IHC. Reduced *ESR1* mRNA expression in total RNA may result from either a lower proportion of ER‐positive tumor cells or decreased ER expression within the ER‐positive tumor cell population. We did not observe a lower percentage of ER positivity by IHC in younger patients, which could reflect fewer ER‐positive cancer cells. Increasing expression of ER in older patients has been mentioned in other studies [23, 24]. In premenopausal women, ER expression fluctuates throughout the menstrual cycle, reaching its peak levels during the follicular phase [25]. ER expression levels in normal breast epithelium tend to increase with advancing age, probably due to a feedback loop as the estrogen level decreases [26]. This also happens in cancers that arise from normal breast in older women. The clinical significance of reduced *ESR1* expression and diminished activity of *ESR1*‐regulated genes in breast cancers diagnosed in young women remains uncertain. Elevated expression of estrogen receptor‐regulated genes is linked to improved overall survival in patients with metastatic breast cancer undergoing hormone therapy [27]. These findings indicate that reduced expression of *ESR1* and its downstream‐regulated genes in breast cancers of young women may reflect decreased responsiveness to endocrine therapy.

In our study, higher *ESR1* mRNA levels in hormone‐positive early breast tumors at the time of diagnosis were significantly associated with increased tumor size, more lymph node involvement, increased tumor stage, and a trend toward more lympho‐vascular invasion. In addition, there was a correlation between *ESR1* mRNA levels and *MKI67* expression, which is associated with tumor cellular proliferation. Breast tumors with higher *ESR1* mRNA levels may be more dependent on *ESR1* and *ESR1*‐regulated genes for growth and progression, and may be more sensitive to endocrine therapy. There was no association between ER IHC and pathological characteristics.

We identified a moderate correlation between *MKI67* mRNA expression and Ki67 IHC. This may be due to translational regulation of Ki67 protein, which is a well‐known mechanism of gene expression regulation in eukaryotic cells. As the IHC tests had been previously performed at local diagnostic laboratories, interobserver variability and technical sensitivity may also have a role. In addition, ki67 IHC is not considered a reliable marker because of the absence of reproducibility [3, 4]. Higher *MKI67* mRNA values were associated with worse clinicopathologic characteristics. Previous studies have shown that both *MKI67* mRNA expression and Ki67 IHC are independent prognostic factors [21, 22].

Our study has limitations: The sample size is small, which may affect the generalizability of the study results.

In conclusion, our study suggests that PCR‐based classification of breast tumors in a central laboratory may be used to confirm the available IHC results performed at local laboratories and add valuable information for patient management. Given the demonstrated prognostic value of mRNA‐based biomarkers in multiple studies, they hold promise for contributing to more standardized approaches in breast cancer management. Additional research is required to evaluate the association of *ESR1*, *PGR*, and *MKI67* mRNA and prognosis markers in this patient cohort.

## Author Contributions

**Hamid Rezvani:** conceptualization, writing – review and editing, project administration, supervision. **Shayan Forghani:** investigation, writing – original draft, methodology, formal analysis, data curation. **Arman Forghani:** investigation, methodology, data curation, formal analysis. **Fatemeh Mahdavi Sabet:** investigation, methodology, formal analysis, data curation. **Atieh Akbari:** conceptualization, writing – review and editing, supervision. **Sanaz Tabarestani:** conceptualization, investigation, supervision, methodology.

## Conflicts of Interest

The authors declare no conflicts of interest.

## Transparency Statement

The lead author, Sanaz Tabarestani, affirms that this manuscript is an honest, accurate, and transparent account of the study being reported; that no important aspects of the study have been omitted; and that any discrepancies from the study as planned (and, if relevant, registered) have been explained.

## Supporting information

Supplementary fig 1.

Supplementary Table 1.

supmat.

## Data Availability

Data underlying the results of this study can be obtained from the corresponding author upon reasonable request.
