# Supplementary figures and images for: Assessment of ESR1, PGR, ERBB2, and MKI67 mRNA in Hormone Receptor‐Positive Early Breast Cancer: A Cross‐Sectional Study
Source: Health Sci Rep. 2025 Jul 15;8(7):e71062. doi: 10.1002/hsr2.71062 (PMC12261032; doi:10.1002/hsr2.71062)

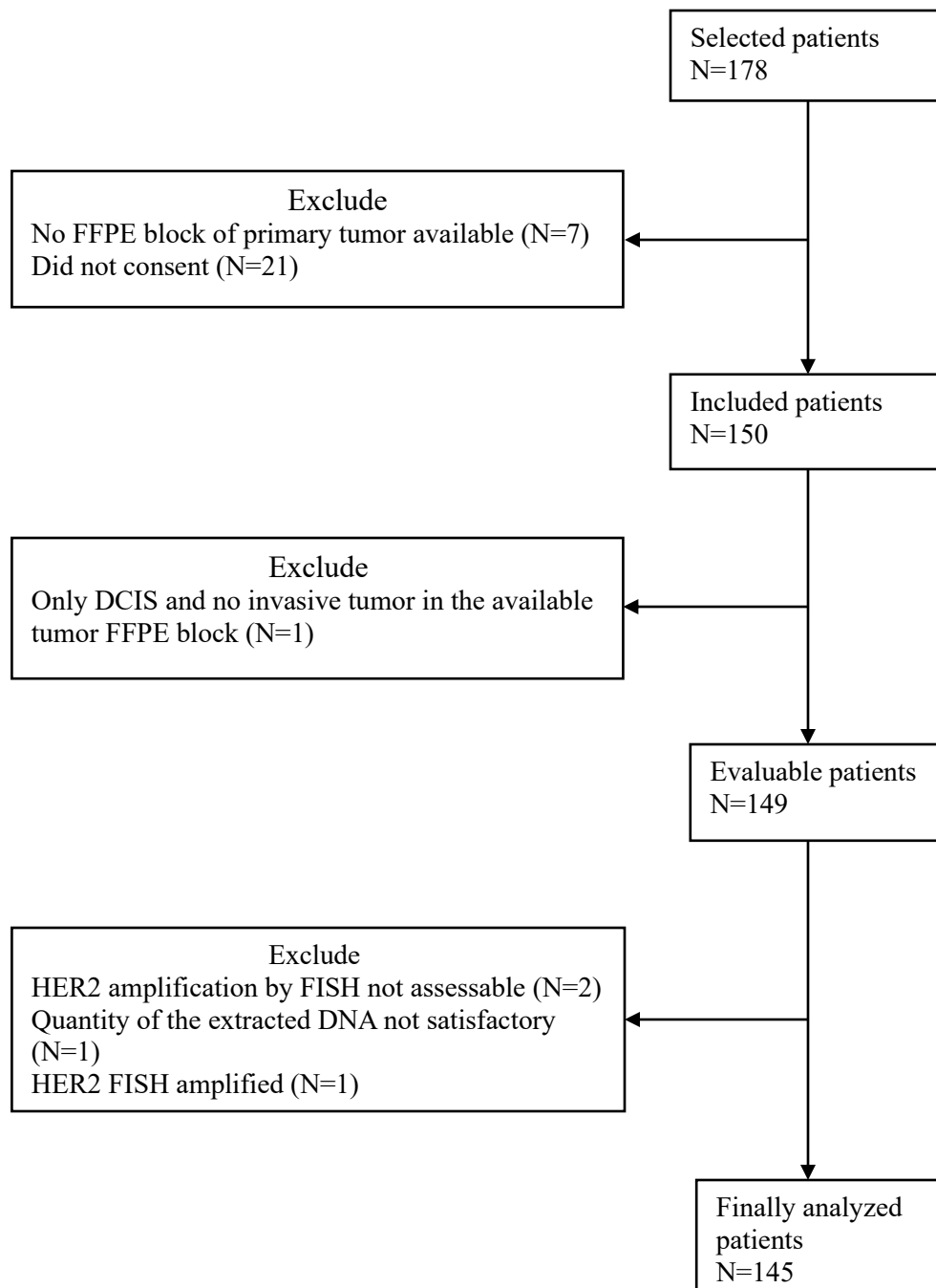

Supplement: Supplementary file 1 — Supplementary fig 1. [file HSR2-8-e71062-s001.pdf]
