# Supplementary material for: Assessment of ESR1, PGR, ERBB2, and MKI67 mRNA in Hormone Receptor‐Positive Early Breast Cancer: A Cross‐Sectional Study
Source: Health Sci Rep. 2025 Jul 15;8(7):e71062. doi: 10.1002/hsr2.71062 (PMC12261032; doi:10.1002/hsr2.71062)
Supplement: Supplementary file 2 — Supplementary Table 1. [file HSR2-8-e71062-s002.docx]

Supplementary Table 1. TaqMan assays for target and reference genes

| TaqMan assay | Gene | Protein | Amplicon size |
| --- | --- | --- | --- |
| Hs01046816_m1 | *ESR1* | ER | 65 |
| Hs01556702_m1 | *PGR* | PR | 77 |
| Hs01001580_m1 | *ERBB2* | HER2 | 60 |
| Hs01032443_m1 | *MKI67* | Ki67 | 66 |
| Hs01060665_g1 | *ACTB* | Actin Beta | 63 |
| Hs00266705_g1 | *GAPDH* | glyceraldehyde-3-phosphate dehydrogenase | 74 |
| Hs00420895_Gh | *RPLP0* | Ribosomal Protein Lateral Stalk Subunit P0 | 76 |
| Hs99999908_m1 | *GUSB* | Glucuronidase Beta | 81 |
| Hs00951083_m1 | *TFRC* | Transferrin Receptor | 66 |

ER: estrogen receptor; PR: progesterone receptor; HER2: human epidermal growth factor receptor 2.
