# Supplementary material for: Assessment of ESR1, PGR, ERBB2, and MKI67 mRNA in Hormone Receptor‐Positive Early Breast Cancer: A Cross‐Sectional Study
Source: Health Sci Rep. 2025 Jul 15;8(7):e71062. doi: 10.1002/hsr2.71062 (PMC12261032; doi:10.1002/hsr2.71062)
Supplement: Supplementary file 3 — supmat. [file HSR2-8-e71062-s003.docx]

**Supplementary fig. 1.** Patient selection diagram. This diagram provides an outline of the patients selected and those excluded due to the lack of consent, tumor FFPE block unavailable, no invasive tumor in the available FFPE block, FISH or RNA extraction not successful, HER2 FISH result positive. FFPE: Formalin-Fixed Paraffin-Embedded; DCIS: Ductal carcinoma in situ; FISH: Fluorescence in situ hybridization.
